# Positive University Environment and Agreeableness as Protective Factors Against Antisocial Behavior in Mexican University Students

**DOI:** 10.3389/fpsyg.2021.662146

**Published:** 2021-07-22

**Authors:** Martha Frías Armenta, Nadia S. Corral-Frías

**Affiliations:** ^1^Department of Law, Universidad de Sonora, Hermosillo, Mexico; ^2^Department of Psychology, Universidad de Sonora, Hermosillo, Mexico

**Keywords:** mood and anxiety, antisocial behaviors, agreeableness, Mexican sample, positive school environment, higher education

## Abstract

Violence in schools is a global issue. Approximately 32% of Mexican students have experienced some form of violence in the school setting in their lives. Previous research has tended to focus on the causes of violence and antisocial behaviors in offenders or adolescent samples and has found evidence to suggest the underlying role of environmental and personal factors. The present study investigates the effect of positive school environment and agreeableness as protective factors against antisocial behaviors in a sample of undergraduate and graduate students (*n* = 304) from northwestern Mexico. Our results demonstrate that a positive school environment has a negative effect on antisocial behaviors via mood and anxiety disorders as well as in interaction with agreeableness, suggesting an interplay between personality and environment. These findings can provide some basis for the development of university programs aimed at fostering positive environments that promote student mental health and protect against antisocial behaviors.

## Introduction

The fear and threat of crime are issues prevalent in much of Latin America. Approximately 24.5 million people were victims of a crime in Mexico alone in 2017, representing 29,746 individuals per 100,000 inhabitants according to the National Survey of Victimization and Perception of Public Safety [Encuesta Nacional de Victimización y Percepción de la Seguridad Pública (ENVIPE)]. Violence in the community often manifests itself in the school setting, where problems such as bullying and threats of violence are highly prevalent (Limber et al., 2018). The Mexican National Commission for Human Rights (CNDH) reports that 32% of students in Mexico have been recipients of some kind of violence in a school setting (National Commission for Human Rights, 2018), although these rates may be higher due to underreporting. Similarly, the Organization for Economic Development and Cooperation estimates that 23% of Mexican students have experienced bullying in some form [Organization for Economic Development and Cooperation (OEDC), 2018]. These high rates in conjunction with research suggesting that violence in schools can negatively impact the emotional, social, and academic development of students as well as school organization and management (Aldridge and McChesney, 2018), highlight the importance of investigating risk and protective factors.

The high prevalence and socioeconomic costs of juvenile delinquency, have led researchers to focus on the causes of violence and general externalizing behaviors characterized by behavioral disinhibition and general acting out behaviors (e.g., substance use, aggression and violence, theft, and property destruction) (Vaughn et al., 2014). Evidence has suggested both environmental and personal factors associated with delinquency and delinquent behavior. A meta-analysis demonstrated negative school climate was associated with problematic student behavior (Reaves et al., 2018) and a systematic review has identified an association between school climate and student mental health (Aldridge and McChesney, 2018). Similarly, personality has been shown to have an effect on antisocial behavior (Vize et al., 2019), which some use as a synonym for a more severe form of externalizing behavior (Liu, 2004). Extant evidence has likewise linked mood and anxiety disorders to antisocial behaviors (Huesmann et al., 2019). Recently, research has begun to extend beyond risk factors and has expanded to examine potential protective factors that may play a role in preventing delinquency such as positive school environment, agreeable personality trait, and mental health.

Key components constituting a positive school environment that promote student social functioning are beginning to emerge. The perception of a fair institutional system and affirmative relationships between peers, students, and instructors represent some of such factors. A longitudinal study in a German secondary school sample demonstrated that prosocial peers in the classroom had a positive influence on classmates (Busching and Krahé, 2020). Further, previous research has suggested that building close student-teacher relationships can protect against the development of internalizing and externalizing disorders in school-aged students (Wang et al., 2013; Olivier and Archambault, 2017). Empirical evidence has likewise linked individual school connection and school engagement with positive student development (Debnam et al., 2014). These studies provide evidence that a positive school environment can lead to favorable effects on the development of social, emotional, and academic competences.

Recent research has also highlighted the influence school positive environments may have on pro-social and antisocial behaviors. A positive school environment has been associated with increased prosocial behavior among students (Luengo Kanacri et al., 2017). Stiff et al. (2019) examined variables related to pro-social behavior in university students and found clarity, affective commitment, empathy, and perspective-taking all emerged in students in the university environment and were related all to pro-social behavior. Pro-social behaviors are also associated with how institutional authorities distribute rewards and punishments (Antrobus et al., 2019), where student perceptions of authority legitimacy may be essential for the appearance of these behaviors. Similarly, instructor support appears to be protective against student academic disengagement (Gasser et al., 2018). On the other hand, perceived unfairness or inconsistency from the authorities in the enforcement of rules have been linked to greater behavioral problems in schools when compared to those with a perceived positive view of authority figures and the discipline system (Brasof and Peterson, 2018). These findings highlight the cyclical nature of interactions between individuals and their environments wherein the interaction of each creates a new socioenvironmental context (Corral Verdugo, 2014).

Personality has also been identified as a potential protective factor against antisocial behavior. Specifically, agreeableness, which is related with motives to uphold interpersonal relations, has been associated directly or indirectly with antisocial behavior (Gleason et al., 2004; Vize et al., 2018). A meta-analysis examining the relationship between the Big Five Factors and antisocial behavior found that agreeableness had the strongest negative association with antisocial behavior (Vize et al., 2019).

Person-environment theory assumes that a behavior can be better predicted if both environmental and personal variables are considered together (van Vianen, 2018). Person-environment theory posits that positive outcomes arise from a good match between personal variables and their environment. Researchers have primarily examined this relationship in the context of academic outcomes. One study analyzed the association between school environment and student personality in an American sample as it relates to student satisfaction and performance and provided evidence that personality, agreeableness, and conscientiousness were associated with satisfaction while school environment was linked to performance (Pawlowska et al., 2014). These results highlight the importance of the interplay between the environment and personality in predicting school satisfaction and performance. Likewise, interactions between environment and personality may predict prosocial behaviors. For instance, the perception of a sense of community and agreeableness were related to prosocial behavior in a study of young participants in Indonesia (Devi et al., 2017). Despite this evidence, few studies have analyzed these variables as protective factors against antisocial behaviors. There is limited research on personality and environment as buffers against antisocial actions and most research has tended to focus on difficult or undesirable early life environments and personality traits more typically associated with risky behavior (Kawamoto, 2020).

Antisocial behavior commonly co-occurs with psychiatric disorders (Tuvblad and Beaver, 2013). Epidemiological studies have suggested that between 12 and 50% of university students may meet criteria for one or more common mental disorders (Blanco et al., 2008b; Hunt and Eisenberg, 2010). Psychiatric disorders in early adulthood are associated with long-term adverse outcomes in later adulthood, including antisocial behaviors. Depression and anxiety have been consistently associated with antisocial behaviors (Huesmann et al., 2019). Depression in early adolescence has been linked to antisocial behavior in adulthood; however, some studies have indicated that depression and anxiety may be outcomes of antisocial behavior (Jolliffe et al., 2019). Kim et al. (2019) also conclude that antisocial behavior could be more a driving mechanism of depression and anxiety than vice versa. Another perspective posits a spiraling model wherein conduct problems during childhood may cause depression and anxiety in adolescence that in turn contribute to greater propensity toward antisocial behavior (Fontaine et al., 2019). In a sample of college students, a history of depression and suicidal behaviors was associated with engagement in delinquent acts (Langhinrichsen-Rohling et al., 2004). Likewise, in a Hispanic sample depression seemed to predict the trajectory of delinquent behavior (Jennings et al., 2019). These studies suggest that antisocial behaviors and mood and anxiety disorders co-occur very frequently and one may lead to the other.

A positive school environment has been linked to increased student wellbeing and a reduction in psychiatric symptoms such as depression and anxiety as well as a reduction in antisocial behaviors. Further, subjective school wellbeing (positive emotions and satisfaction with the school) has been linked to prosocial behavior (Chen et al., 2020). For instance, teacher implementation of justice in the classroom was positively related to emotional engagement, classroom connectedness and these in turn predicted student social functioning and mental health (Mameli et al., 2018). A longitudinal study of secondary school students, which investigated social climate, learning opportunities, fairness and clarity of rules, safety and depressive symptoms in adolescents, suggested that a better socio-educational environment reduced depressive symptoms at later stages of life (Brière et al., 2013). Similarly, students belief in a just world was related to well-being in undergraduate students (Yu et al., 2017). Likewise, middle school students that perceived instructors as just appear to be less likely to participate in violent behaviors (Resh and Sabbagh, 2014).

**FIGURE 1:**
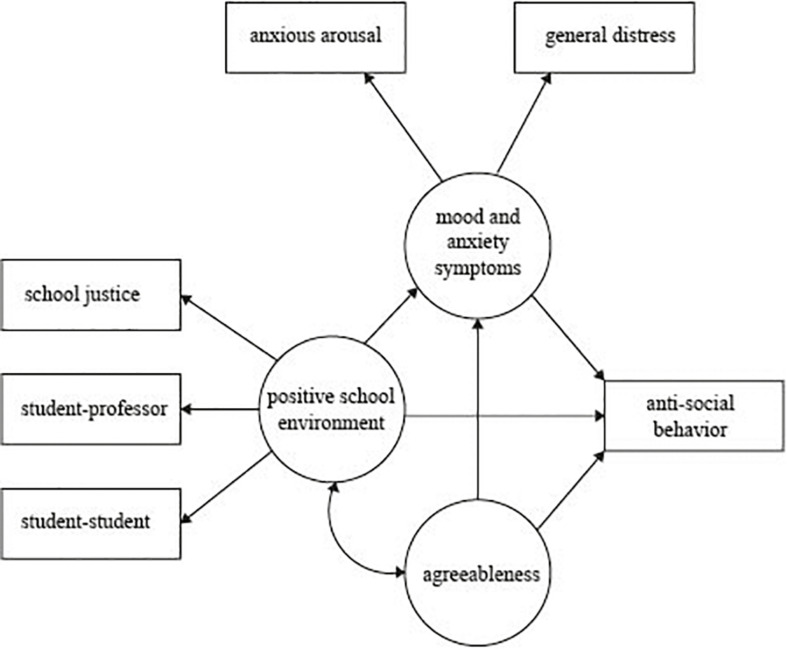
Hypothetical model of positive school environment and agreeableness as protective factors for antisocial behaviors. The model predicts that a positive school environment will have a direct effect on mood and anxiety disorders and antisocial behaviors and indirect effect on antisocial behaviors through mood and anxiety disorders. Agreeableness will have a direct effect on antisocial behaviors and will covary with positive school environment.

Protective factors against antisocial behavior have primarily focused on offender or adolescent samples. However, some antisocial behaviors are common among the general population (Blanco et al., 2008a; Grant et al., 2011) and specifically in university students (e.g., stealing and driving violations) (Grant et al., 2016). While it is important to identify the underlying factors that lead to antisocial behavior, it is likewise critical to examine factors that may protect against such behaviors across contexts. There are limited studies of antisocial behavior among university students and fewer have investigated the factors that may protect against these behaviors. Given previous evidence, the aim of this study was to investigate the effect of agreeableness and positive school environment as potential factors that protect against antisocial behavior in university students as well as possible relationships with mood and anxiety disorders. See Figure 1 for the proposed model. Given the previously stated literature, we predict that a positive school environment will have a direct effect on mood and anxiety disorders and antisocial behaviors. Likewise, we hypothesize an indirect effect on antisocial behaviors through mood and anxiety disorders. In addition, we expect personality will also have a direct effect on antisocial behaviors and will covary with positive school environment.

## Materials and Methods

### Participants

Participants included 304 undergraduate (34 different majors) and graduate students from various universities in a northwestern city in Mexico. Graduate students were included in the sample in an attempt to represent the entirety of the student population in a university. In congruence with the population of most universities, our sample included a small portion of graduate students (9.2%) and no differences were found in any of the studied variables between graduate and undergraduate students. An educational attainment question indicated that 69% of participants completed high school, 24% undergraduate school, 3.3% master’s degree, and 0.3% Ph.D. Approximately 30% reported working in addition to their academic pursuits. Most of the participants identified as female (67% as female, 29% as a male, 1% non-binary, and 0.6% preferred not to answer). Age ranged from 17 to 44, where the mean was 21.63 years (SD = 3.91). Most participants reported being single (71.8% single 5.8% married, and 20% living with a partner).

### Instruments

Participants were asked several demographic questions assessing age, education, and gender. Additionally, we used scales to assess antisocial behavior, agreeableness, depression, anxiety, and school environment.

*Antisocial behavior* was measured using 21 previously translated items from the Normative Deviance Scale (NDS) (Vazsonyi et al., 2001). Participants responded if they generally took action, using 6-points scale (1 = never, 2 = 1–2 times, 3 = 3–5 times, 4 = 6–10 times, 5 = 11–20 times, and 6 = more than 20 times), in behaviors that go against social norms, independently of the definitions of crime based on General Theory of Crime (Gottfredson and Hirschi, 1990). Some examples of the items are: “Intentionally damaged or destroyed property?”, “Let the air out of the tires of a car or bike?” “Cheated on school/college/university tests (e.g., cheat sheet, copy from neighbor, etc.)?” This scale showed acceptable internal consistency reliability (α = 0.87) in our study.

The *positive school environment* factor was assessed via three related constructs: institutional justice, student-professor relationship, and student-student relationship. The scale has been previously validated in Mexico (Valenzuela et al., Submitted). Students expressed if they, in general, felt justice in their university and had a good relationship with their professors as well as other students. The *student-professor relationship* subscale included seven items, where participants responded using a 5-point Likert-style scale, (1 = strongly disagree to 5 = strongly agree), and assessed student perception of the socioemotional competences of professors and how well they got along with them. This subscale showed acceptable internal consistency reliability (α = 0.85) in this study. Some examples of items are “very frequently it is difficult for teachers and professors to understand each other (reversed keyed item)”, “students can give their opinion in the establishment of rules in the classroom” The *student-student relation* subscale had seven items in 5-point Likert (1 = strongly disagree to 5 = strongly agree) that assessed the relationship between students in the school. This scale showed acceptable internal consistency reliability (α = 0.84) in this study. Some examples of the items are: “There is a lot of anger/dislike/resentment among my peers (reversed keyed item),” “Classmates feel very close to each other,” “My classmates and I share interests and hobbies.” To assess *institutional justice*, we used twelve items, 5-point Likert-style (1 = strongly disagree to 5 = strongly agree) with questions regarding their perception of justice, access to student services, and involvement in the decision-making process. This scale showed acceptable internal consistency reliability (α = 0.76) in this study. Examples of the items are: “Each member of the university can give their opinion about important decisions,” “All members of the university have confidence in the authorities,” “The authorities are impartial in making decisions.”

*Mood and anxiety symptom* were assessed using the mini-MASQ (Casillas and Clark, 2000), which included twenty-six, 5-point Likert-style items (1 = nothing to 5 = extremely) with three sub-scales: general distress (8 items), anhedonic depression (8 items), and anxious arousal (10 arousal). For this study, we used only general distress and anxious arousal subscales, which included items such as “I felt depressed,” “I felt useless,” and “I felt tense” during last week. This scale was previously validated in a Mexican population (Corral-Frías et al., 2019). These subscales showed acceptable internal consistency reliability (GD: α = 0.91; AA: α = 0.89) in this study.

*Agreeableness* was measured using a translation of the Faceted Inventory of the Five-Factor Model (FI-FFM) designed to measure the facets of the Big Five traits consisting of 207, 5-point Likert-style items (Watson et al., 2017). For this study, we only used the 48 agreeableness items. Participants answered items such as “Whenever I can, I cooperate with other people” and “I always try to consider the needs of others” are some examples of items. This scale was previously validated in Mexican samples (Corral-Frías et al., in preparation). This scale showed acceptable internal consistency reliability (α = 0.84) in our study.

### Procedure

Students were recruited using promotional materials published and posted at different campuses as well as oral announcements in classrooms and link sharing in associated Facebook pages. Prior to participation, the objectives of the study were explained and students were informed that their participation was voluntary and that they could discontinue their participation at any time. After this explanation, students were given the opportunity read the online informed consent form alone. Students began their participation in the study in a psychology lab after signing the form electronically. Participants completed the questionnaires using a tablet or a laboratory computer using the Qualtrics software platform. This study was part of an ongoing larger project, and this was the first of the four visits across the academic year. Questionnaire completion time was approximately 30 min. After completion, each student received economic compensation for their participation ($200 Mexican pesos, ∼$10 USD). The study was approved by the University of Sonora Ethics Committee prior to implementation. All procedures contributing to this work comply with the ethical standards of the relevant human experimentation guidelines and in congruence with the Helsinki Declaration of 1975, as revised in 2008.

### Data Analysis

Univariate statistics were calculated using SPSS v25, means and standard deviations for continuous variables and frequencies for categorical variables. All instruments demonstrated acceptable internal consistency reliability. Table 1 show descriptive statistics and alpha coefficient scores for each of the scales utilized.

**TABLE 1:** Reliability and univariate statistics of utilized scales (scale range of responses: 1–5).

*Scale (number of items)*	*Min*	*Max*	*Mean*	*Standard deviation*	*Skewness*	*Coefficient alpha*
**Agreeableness (42)**	**2.23**	**4.40**	**3.48**	**0.40**	−**0.37**	**0.84**
**Positive University Environment (36)**	**2.03**	**4.73**	**3.46**	**0.43**	−**0.28**	**0.88**
Institutional justice (12)	1.83	4.50	3.22	0.49	–0.30	0.76
Student-student relationship (12)	1.70	5.00	3.65	0.58	–0.39	0.84
Student-professor relationship (12)	1.50	5.00	3.50	0.61	–0.49	0.85
**Mood and Anxiety Symptoms Questionnaire (18)**	**1.00**	**4.85**	**2.01**	**0.86**	**0.95**	**0.94**
General distress (8)	1.00	5.00	2.25	1.03	0.75	0.91
Anxious arousal (10)	1.00	4.70	1.77	0.80	1.32	0.89
**Antisocial behaviors (21)**	**1.00**	**2.10**	**1.32**	**0.25**	**1.54**	**0.87**

*Bolded values correspond to statistics for scales, whereas unbolded are for each of subscales.*

Antisocial behavior scores were winsorized to maintain variability while limiting the influence of extreme outliers before being analyzed in SPSS. Scores that exceeded the mean plus 2 standard deviations were set to that value (10 participant scores had scores higher than 2.10 and were set to 2.10).

The proposed theoretical model was probed using structural modeling via EQS (Bentler, 2006). Given the normal distribution of the data (Mardia-based Kappa = 0.60) we used the ML method (Bentler, 2006). To determine the relevance of the model we considered χ^2^ for robust variance (Asparouhov and Muthen, 2013) as an indicator of statistical goodness of fit, where the value should be low and non-significant (*p* > 0.05). Similarly, Bentler Bonett Normed Fit Index (BBNFI), Bentler Bonett Non-Normed Fit Index (BBNNFI), Comparative Fix Index (CFI), were used as goodness of fit indices, where we sought values 0.90 and higher (Bentler, 2006). To measure the reasonable approximation error (RMSEA) we used the square error index which value should be below 0.08 (Zhang and Savalei, 2016). Finally, given the observed interaction between personality and environment, an exploratory regression-based moderation model was tested using the PROCESS macro for SPSS (Hayes, 2017) to examine interactive effects of a positive university environment and agreeableness on antisocial behaviors.

## Results

**FIGURE 2:**
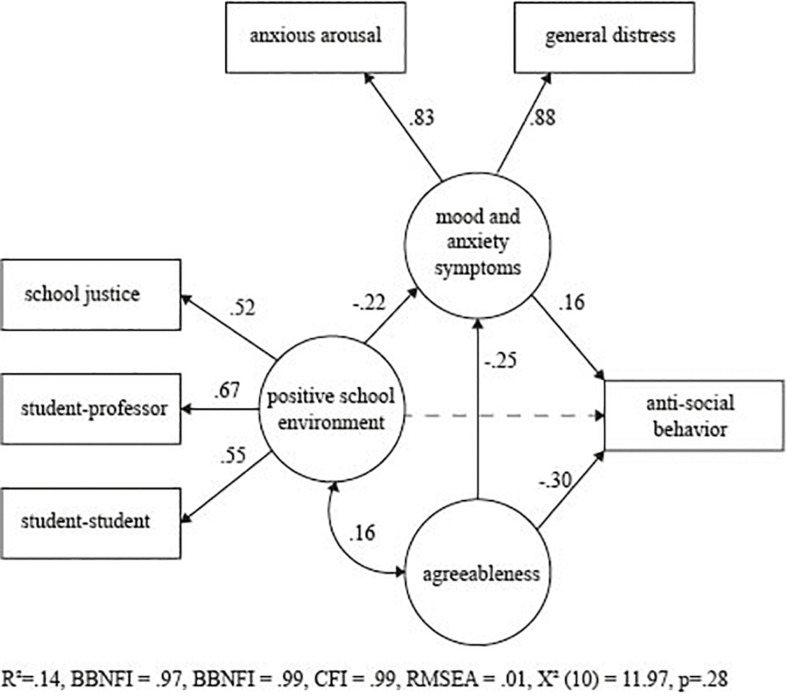
Structural equations model of protective enviromental and personality factors for antisocial behavior. All factor loadings, structural coefficients, and covariances were significant (*p* < 0.05); except one marked with dotted line. The model demonstrated acceptable Goodness of Fit [χ^2^ (10) = 11.97, *p* = 0.28; BBNFI = 0.97, BBNNFI = 0.99, CFI = 0.99, RMSEA = 0.01].

**FIGURE 3:**
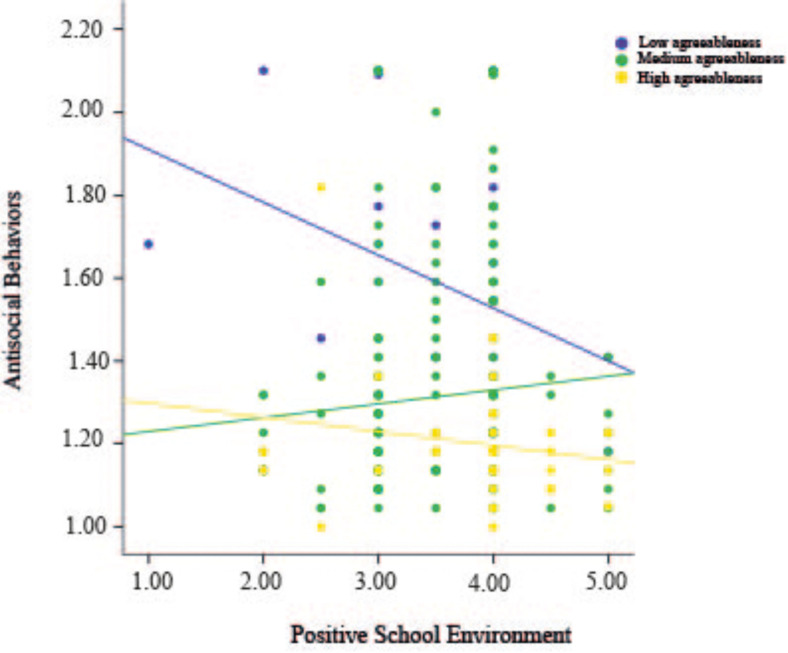
Regression model measuring the moderating role of agreeableness between positive environment and antisocial behaviors in Mexican university students. A significant interaction between agreeableness and positive environment was found (*R*^2^ = 0.41, Δ*R*^2^ = 0.01, *b* = 0.08, *p* = 0.04).

Table 1 shows descriptive statistics of each of the instruments utilized in the study. Regarding our main variable of interest, most participants reported committing antisocial behaviors on average at least once or twice (95.3%). However, a low proportion stated being involved in such behaviors more than three times (4.7%). Figure 2 shows the structural equation model confirming the coherence of the theoretical specified factors and its relationships. The Positive School Environment (PSE) factor was formed by three variables: student-professor relationship (λ = 0.67), student-student relationship (λ = 0.55), and institutional justice (λ = 0.52). The mood and anxiety factor included of general distress (λ = 0.88) and anxiety (λ = 0.83). The structural equations model demonstrated a direct and negative effect of agreeableness on antisocial behavior (structural coefficient −0.30) and mood and anxiety factors (structural coefficient, −0.25). PSE had a negative effect on mood and anxiety (structural model −0.22); however, a direct effect on antisocial behavior was not significant. Finally, mood and anxiety had a direct and positive effect on antisocial behavior (structural coefficient 0.16). The model demonstrated acceptable Goodness of Fit [χ^2^ (10) = 11.97, *p* = 0.28; BBNFI = 0.97, BBNNFI = 0.99, CFI = 0.99, RMSEA = 0.01] suggesting that the theoretical model adjusted to the data. This model predicted 14% of the variance of antisocial behaviors Given that we found significant associations between personality and school environment in the SEM we wanted to probe if there would be interaction to predict antisocial behavior (i.e., moderating effect). We found significant interaction between agreeableness and positive environment (*R*^2^ = 0.41, Δ*R*^2^ = 0.01, *b* = 0.08, *p* = 0.04), where those who reported low agreeableness and negative school environment reported increased antisocial behavior (see Figure 3). Johnson-Neyman *post hoc* analyses showed that this effect is significant at low levels of agreeableness (*t* = −2.08, *p* = 0.03) but not at higher levels (*t* = 0.32, *p* = 0.74).

## Discussion

Our study is unique in that, to the authors’ present knowledge, this is one of the first investigations to assess possible protective environmental and personal variables against antisocial behaviors in the university context in Latin America. Our results demonstrate that a positive school environment has a negative effect on antisocial behaviors via mood and anxiety disorders and in interaction with agreeableness in a sample of Mexican university students. We discuss the implications of our findings.

Our model demonstrated a negative association between positive school environment and mood and anxiety symptoms. This is in congruence with previous literature suggesting a significant role of the school environment of mood and anxiety symptoms (Dunn et al., 2015). A recent study from Uganda found that children with low school connectedness in addition to experiences of violence from school staff and other students had higher probability of reporting mental health symptoms (Thumann et al., 2016). Moreover, longitudinal research has demonstrated that school environment has an effect on depressive symptoms in students in Canada (Brière et al., 2013), China (Nie et al., 2020), and Sweden (László et al., 2019) suggesting that school environment has long-term effects on student mental health across various countries.

As expected, our results provide evidence of an association between mood and anxiety symptoms and antisocial behaviors. Given that our sample is not longitudinal we are unable to make any assumptions about the directionality of this association. However, these findings are in congruence with previous evidence demonstrating internalizing symptoms to be significantly associated to externalizing behaviors (Cole and Carpentieri, 1990; Eisenberg et al., 2001; Ormel et al., 2005; Bornstein et al., 2010). We found that those with greater incidence of self-report mood and anxiety symptoms also reported committing antisocial acts. Our findings are significant because in congruence with a longitudinal investigation found that as age increased internalizing symptoms accounted for a greater degree of externalizing problem variance (Perle et al., 2013) providing an avenue for reducing antisocial behaviors in adulthood through the early treatment of internalizing problems.

We did not find a direct association between positive school environment and antisocial behaviors. Previous studies in Mexican high school students found that some school context variables (equal treatment by teachers but not teacher bonding) were relevant in predicting involvement in school vandalism (Vilalta and Fondevila, 2017). Our lack of association may be due to age differences in the sample (e.g., high school teachers spend more time with students) or the inclusion of other subscales in the positive environment construct in this study (i.e., peer relationship and school justice). Although we did not find a direct association, we did find that the interaction between positive school environment and personality predicted antisocial behaviors. This is congruent with the person-environment theory (van Vianen, 2018) and recent empirical evidence in prosocial literature. A study in Indonesia, demonstrated that sense of community and agreeableness were related to prosocial behavior (Devi et al., 2017). However, to the authors’ knowledge, this is one of the first investigations that provide evidence toward an interaction between positive school environment and agreeableness to predict reduced antisocial behaviors in a university sample. Our data suggest that agreeableness is protective in all environments. However, in those with low agreeableness providing a positive environment reduces risk for antisocial behaviors. This evidence, which should be further tested for replication, can lead to important preventative interventions that can significantly reduce risk.

This study is not without limitations. Our investigation was cross-sectional and thus we cannot make assumptions of causality. Furthermore, our sample (*n* = 304) had enough power to detect associations but may be limited in other ways. For example, we included students from just one northwestern city in Mexico and thus it is not representative of the Mexican higher education population. Nevertheless, our sample did include both private and public institutions representing students from diverse backgrounds and academic focus. The sample was predominantly female and externalizing symptoms tend to be more prevalent in males, which may have had an effect on our results (Leadbeater et al., 1999; Daughters et al., 2009). Further, the sample was a convenience sample and may thus include students who are inclined to participate in studies. This may have biased our sample by including more agreeable people. In addition, our results are derived from a low antisocial behavior reporting sample, University students (see Table 1). Thus, it is crucial to have follow-up studies, especially in larger community samples to test the replicability of our results. Finally, although we included some important variables, our model only explained 15% of the variance, suggesting there are other factors that need to be included to improve the model. Early-life and current stress as well as neighborhood and family environment are other important factors that may influence the appearance of antisocial behaviors. Given these limitations, future studies should include longitudinal data and a larger more representative sample.

Despite these limitations, our results suggest that positive school environments can be protective against psychopathology symptoms and thus may reduce the appearance of antisocial behaviors in university students. Future studies should seek to replicate these findings in other cultures and countries. New worldwide initiatives such as the Psychological Science Acceleration (Moshontz et al., 2018) and Psi Chi’s Network for International Collaborative Exchange (Psi Chi, 2020) are gaining momentum to probe cultural variance/invariance as it relates to psychological phenomenon (Jones et al., 2021). Further, there have been calls to study student misconduct internationally (Pascal et al., 2019). Further, these questions should be addressed in longitudinal designs to truly test protective nature of these variables. As stated in the methods, the present study is part of a larger investigation which followed students for a full academic year. We plan to report data from follow up visits, however, the true causal nature of these protective factors can only be elucidated by following participants from an early age.

Our findings can provide helpful information for the development of possible interventions. As the main objective of this study was to investigate possible protective factors against antisocial behaviors, the knowledge gained here provides some insights as to potential preventative efforts. For example, given that the professor-student relationship is protective an initiative workshops to train professors to improve socio-emotional competence may improve student-professor relationships and thus student mental health and potential behavior problems. Some parent-focused interventions have shown to be effective in Mexico (Amador Buenabad et al., 2020). Stemming from studies at lower educational levels, researchers have suggested that providing professors with tools to develop better relationships with students may improve the learning process, increase engagement, as well as serve a protective role (Wang et al., 2013; Olivier and Archambault, 2017; Cheon et al., 2019; Prewett et al., 2019).

Given our results, and in congruence with previous research, we suggest seeking improvement in school and classroom justice at the university level through professor training. Training professors to better communicate and respond to student needs may improve perception of justice. Previous studies have shown that assertive and responsive behaviors as well as demonstrating content expertise, affection toward students, and verbal fluency can lead to increased perceptions of fairness (McCroskey et al., 2004; Myers et al., 2009). Indeed, empirical research found an association between perception of fairness and classroom justice (Chory, 2007) wherein the author suggests that the perception of professor justice and credibility may protect against the appearance of antisocial behavior.

We further suggest school sanctioned bonding activities and or workshops to improve student-student relationships. Previous empirical research has highlighted the importance of nurturing peer relationships for a positive experience during university education (Maunder, 2018). Although peer network intervention studies have focused on school-aged children (Miller et al., 2017), students with disabilities (Asmus et al., 2017), and autism diagnosis (Odom, 2019), a recent randomized experimental study found that using a closeness-induction task improved peer connections among undergraduate students (Rasco et al., 2020). A previous review posited “the effectiveness of compassion education and training approaches in establishing a safer, healthier, happier, and more inclusive educational learning environment leading to enhanced prosocial behaviors and positive mental health” (Tendhar and Bueno de Mesquita, 2020). A comprehensive approach to the implementation of these types of policies may improve not only student mental health but may also reduce student antisocial behavior and provide a more positive school environment for all.

## Data Availability Statement

The raw data supporting the conclusions of this article will be made available by the authors, without undue reservation.

## Ethics Statement

The studies involving human participants were reviewed and approved by the University of Sonora Ethical Committee. The patients/participants provided their written informed consent to participate in this study.

## Author Contributions

Both authors listed have made a substantial, direct and intellectual contribution to the work, and approved it for publication.

## Conflict of Interest

The authors declare that the research was conducted in the absence of any commercial or financial relationships that could be construed as a potential conflict of interest.
